# Intra-pulmonary migration of a clavicle osteosynthesis pin: a case report

**DOI:** 10.1186/s13256-024-04369-7

**Published:** 2024-03-28

**Authors:** Kaouther Ben Amara, Sarra Zairi, Bechir Ben Radhia, Mahdi Abdennadher, Hazem Zribi, Adel Marghli

**Affiliations:** 1 Thoracic Surgery Department, Abderrahmen Mami University Hospital-Ariana, Faculty of Medicine of Tunis, University of Tunis El Manar, Ariana, Tunisia; 2 Cardio-Thoracic Surgery Department, University Sahloul Hospital-Sousse, Faculty of Medicine “Ibn El Jazzar” of Sousse, Sousse, Tunisia

**Keywords:** Clavicle fracture, Kirschner pin, Foreign-body migration, Lung injury

## Abstract

**Background:**

Fractures of the clavicle are common injuries, which often require reduction and internal fixation. Although Kirschner pins have been commonly used to treat these fractures with good results, migration of these devices may result in severe internal lesions.

**Case presentation:**

We report herein the case of 61-year-old man, who presented for intrapulmonary migration of a Kirschner pin, 25 years after closed reduction and fixation of a clavicle fracture.

**Conclusion:**

Migration of an osteosynthesis pin can be lethal. Patients with osteosynthesis pins, should have a regular follow, until the removal of the wires.

## Background

Fractures of the clavicle are common injuries. Most of them are successfully managed with conservative treatment. However, osteosynthesis with internal fixation may be required in some displaced fractures to avoid nonunion or malunion with secondary poor shoulder function [1]. Although Kirschner pins (K-pins) have been commonly used to treat these fractures with good results, secondary migration of these devices may result in serious vascular or visceral injuries [1–6]. We report herein the case of an intrapulmonary K-pin migration.

## Case presentation

**Fig. 1:**
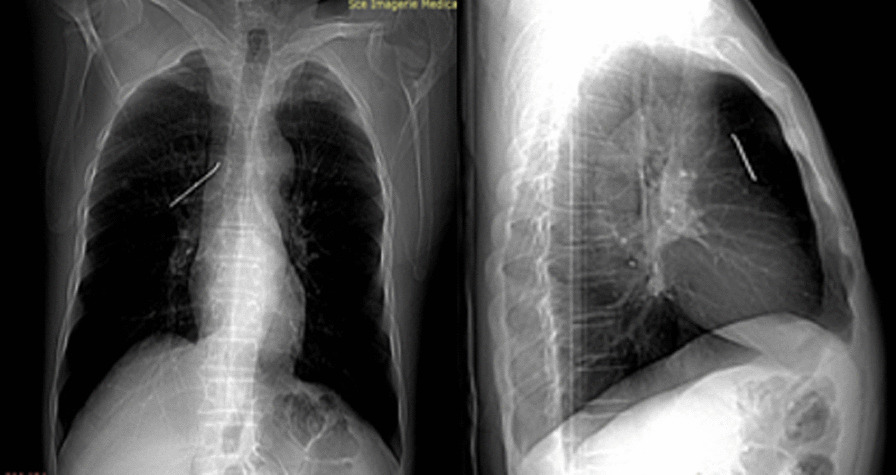
Chest X ray showing a Kirschner pins in the right upper lobe

**Fig. 2:**
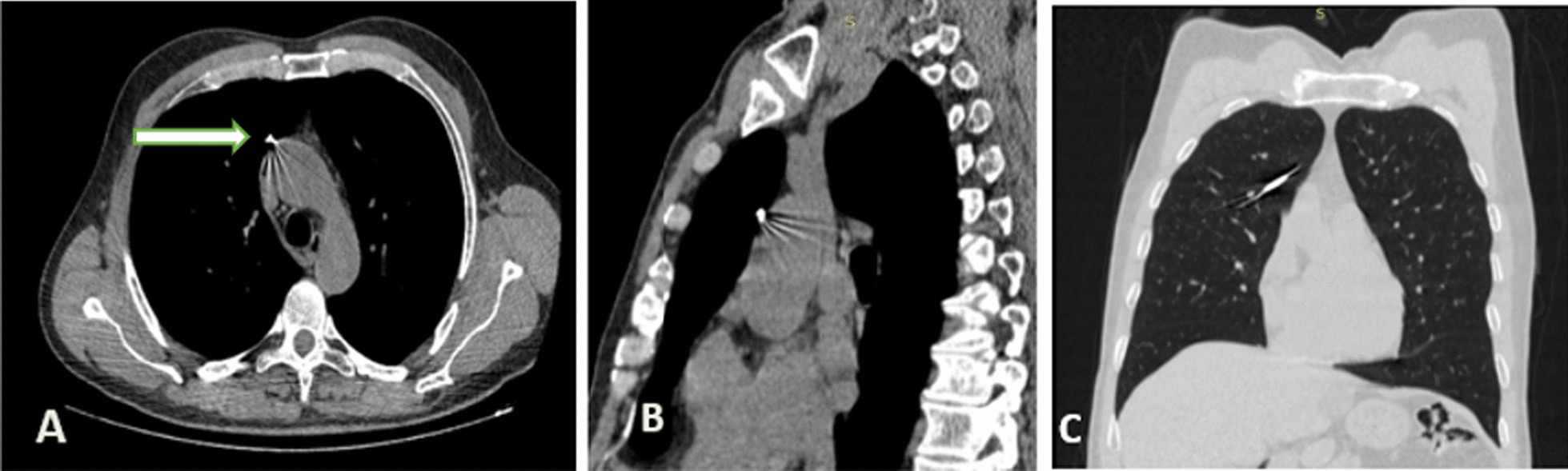
**A**–**C** Chest Computed tomography showing the wire in the upper right lobe adjacent to ascending aorta (white arrow shows well the pin)

**Fig. 3:**
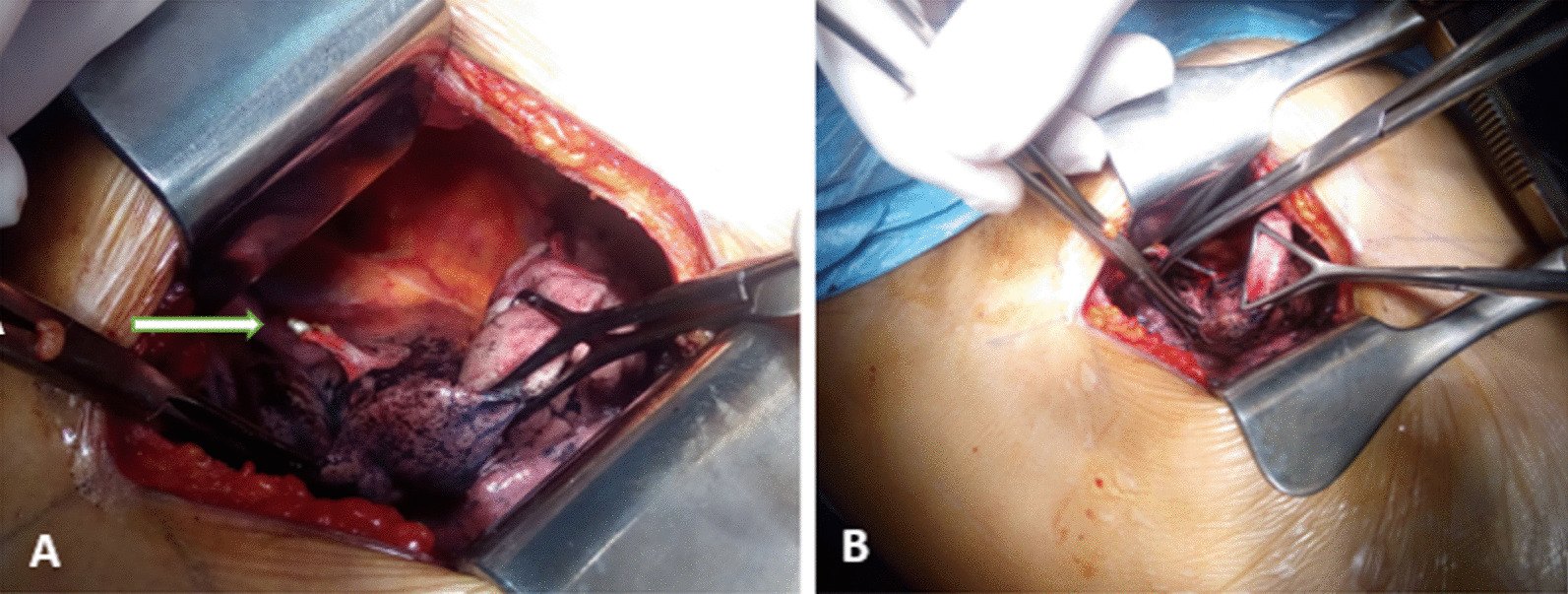
Intra-operative images, Kirschner pins sitting seated in the mediastinal surface of the right upper lobe (**A**), removal of the pin (**B**) (white arrow shows well the pin)

A 61-year-old smoking man presented to our outpatient clinic for recurrent tingling right chest pain. He had no comorbidities but, reported a right clavicle fracture which warranted surgical osteosynthesis, with internal fixation with a K-pin 25 years ago. Physical examination was normal. Chest X ray showed a K-pin in the right upper lobe (Fig. 1). The patient was unaware of the need to remove the pin after consolidation; therefore, he didn’t comply with follow-up. Chest CT with contrast enhancement showed a well-consolidated clavicle. No lesion of the great vessels or pneumothorax were shown. The pin’s tip was in close contact with the ascending aorta (Fig. 2). Given the potential involvement of the mediastinum, urgent surgical removal was decided through a lateral mini thoracotomy. The pin’s tip was retrieved in the surface of the right upper lobe without significant mediastinal adherence with the ascending aorta. A pneumotomy was performed above the pin’s tip, which was grasped with an indented forceps. It was gently mobilized laterally and then carefully pulled out, without secondary bleeding (Fig. 3). The parenchyma was tested for air leak. After thorough washing, the pneumotomy was closed with interrupted resorbable sutures and pleural cavity was closed on a chest tube. The postoperative course was uneventful. The patient was discharged 2 days after surgery. Further follow-up was uneventful.

## Discussion

We reported the case of 61-year-old man who presented for an intrapulmonary migration of a K-pin, 25 years after a closed reduction and fixation of a clavicle fracture.

The migration of K-pins or metallic wires had been scarcely reported in literature. Lyons and Rockwood described 49 cases of K-wire migration in 47 patients, reported between 1943 and 1981 [7]. A recent review of the literature reported 68 cases of intra-thoracic migration. In most of these cases, migration was early. However, a great variability in the interval between positioning and migration of the K-pin has been reported, varying from 1 day to 21 years [2, 8]. In our case, migration was discovered 25 years later. Despite many attempts to explain the peculiar phenomenon, the exact reason remains unclear. Several factors might contribute to migration, such as muscle action, respiratory movements, intra-thoracic negative pressure, regional bone resorption, gravitational forces and the great range of motion of the upper extremity [6]. Some authors reported axillary migration with or without nervous lesions. Intra-thoracic migration is often followed with subsequent complication such as pneumothorax, hemothorax, lung consolidation, or hemoptysis. Migration to the lung had been mainly revealed with hemoptysis or pneumothorax [1, 4]. Fatal cardiac and vascular perforation [5] with hemothorax, collapse and pericardial tamponade, had also been described. Intra-abdominal migration through the diaphragm with or without intestinal perforation was also reported in 2 cases [2]. A variety of signs and complications have been described as a result of intra-thoracic migration such as pain, dyspnea, hemoptysis, anemia, subcutaneous emphysema, respiratory distress, and cardiac tamponade [4]. K-pin migration could be completely asymptomatic, dangerously delaying the diagnosis [2]. The type of the wire used, smooth, threaded or bent, did not impact the occurrence of migration [7].

Several precautions should be taken if K-pins are to be used for internal fixation of shoulder girdle fractures and dislocation. To prevent potential migration, it is highly recommended to bent sufficiently the extremities of the wire to involve the periosteum, or positioning the wire with percutaneous technique, avoiding, if possible, anterograde wire positioning. For tuberosity fixation, the use of a cannulated screw should be considered [2]. During fixation, K-pin positioning should be accurate, and, after pinning, hardware stability should be always checked through dynamic maneuvers. Pinning techniques should be carefully discussed especially in cases of elder patients with presumably low bone quality and low compliance to the immobilization regimen [2, 9]. Moreover, the patient must receive close clinical and radiographic follow-up until the K-pins are removed [9, 10]. The wires must be withdrawn when the desired therapeutic effect has been attained [10]. The surgeon must be aware of the risk of devastating organ damages. Reported complications of fractures, or shoulder girdle dislocation, particularly those arising at the sterno-clavicular joint, should be kept in mind.

In addition, displacements of considerable distance may occur in only a few hours after the diagnosis [1]. Our patient was lost to follow-up because he was unaware of the need to remove the pin.

If any sign of migration is detected, an X-ray and a CT scan should be performed. Surgical removal should be promptly performed, to prevent further complications. In the case of intra-thoracic migration, sternotomy [3] or thoracotomy [5] have been used by different authors. Sternotomy may be considered more appropriate in emergent conditions or in the presence of a cardiac or an intra-pericardial vascular injury [8]. A minimally invasive approach, such as video-assisted thoracoscopy may be useful for retrieving and removing an intrapulmonary migrated pin [11]. In our patient, we used a mini-lateral thoracotomy.

## Conclusion

Although our patient underwent a successful removal of the wire and recovered uneventfully, migration of an osteosynthesis pin can be lethal. Patients with osteosynthesis pins, should have a regular follow, until the removal of the wires.

The surgeon must explain to the patient the importance of the regular follow-up, in order to detect any complication as soon as possible.

## Data Availability

Not applicable.

## Abbreviations

K-pins: Kirschner pins
CT: Computed tomography

## References

[1] Cameliere L, Rosat P, Heyndrickx M, Le Rochais J-P, Icard PJAC, Annals T (2013). Migration of a Kirschner pin from the shoulder to the lung, requiring surgery. Asian Cardiovasc Thorac Ann.

[2] Cerruti P, Mangano T, Giovale M, Repetto I (2016). Early asymptomatic intrathoracic migration of a threaded pin after proximal humeral osteosynthesis. Int J Shoulder Surg.

[3] Janssens de Varebeke B, Van Osselaer G (1993). Migration of Kirschner's pin from the right sternoclavicular joint resulting in perforation of the pulmonary artery main trunk. Acta Chirurg Belgica.

[4] Mellado J, Calmet J, García Forcada I, Saurí A, Giné J (2004). Early intrathoracic migration of Kirschner wires used for percutaneous osteosynthesis of a two-part humeral neck fracture: a case report. Emerg Radiol.

[5] Nordback I, Markkula HJ (1985). Migration of Kirschner pin from clavicle into ascending aorta. Acta Chirurg Scand.

[6] Wang S-Q, Gao Y-S, Mei J, Ni M, Wang J-Q, Zeng Z-L (2010). Migration of a broken Kirschner pin into thoracic spinal canal 4 years following internal fixation of a clavicle fracture. Eur J Orthopaed Surg Traumol.

[7] Kumar P, Godbole R, Rees GM, Sarkar PJ (2002). Intrathoracic migration of a Kirschner wire. J Roy Soc Med.

[8] Venissac N, Alifano M, Dahan M, Mouroux J (2000). Intrathoracic migration of Kirschner pins. J Roy Soc Med.

[9] Fransen P, Bourgeois S, Rommens JJAOB (2007). Kirschner wire migration causing spinal cord injury one year after internal fixation of a clavicle fracture. Acta Orthopaed Belgica.

[10] Nakayama M, Gika M, Fukuda H, Yamahata T, Aoki K, Shiba S (2009). Migration of a Kirschner wire from the clavicle into the intrathoracic trachea. Ann Thorac Surg.

[11] Calkins CM, Moore EE, Johnson JL, Smith WR (2001). Removal of an intrathoracic migrated fixation pin by thoracoscopy. Ann Thorac Surg.

